# A near chromosome-level assembly of the serpentine endemic columbine, *Aquilegia eximia*

**DOI:** 10.1093/jhered/esaf035

**Published:** 2025-06-10

**Authors:** Jason Johns, Merly Escalona, Courtney Miller, Noravit Chumchim, Oanh Nguyen, Mohan P A Marimuthu, Samuel Sacco, Colin Fairbairn, Eric Beraut, Erin Toffelmier, H Bradley Shaffer, Scott Hodges

**Affiliations:** Department of Ecology, Evolution, and Marine Biology, University of California, Santa Barbara, Santa Barbara, CA, United States; Department of Biomolecular Engineering, University of California, Santa Cruz, Santa Cruz, CA, United States; UCLA La Kretz Center for California Conservation Science, Institute of the Environment and Sustainability, University of California, Los Angeles, Los Angeles, CA, United States; DNA Technologies and Expression Analysis Core Laboratory, University of California Davis, Davis, CA, United States; DNA Technologies and Expression Analysis Core Laboratory, University of California Davis, Davis, CA, United States; DNA Technologies and Expression Analysis Core Laboratory, University of California Davis, Davis, CA, United States; Department of Ecology and Evolutionary Biology, University of California, Santa Cruz, Santa Cruz, CA, United States; Department of Ecology and Evolutionary Biology, University of California, Santa Cruz, Santa Cruz, CA, United States; Department of Ecology and Evolutionary Biology, University of California, Santa Cruz, Santa Cruz, CA, United States; UCLA La Kretz Center for California Conservation Science, Institute of the Environment and Sustainability, University of California, Los Angeles, Los Angeles, CA, United States; UCLA La Kretz Center for California Conservation Science, Institute of the Environment and Sustainability, University of California, Los Angeles, Los Angeles, CA, United States; Department of Ecology, Evolution, and Marine Biology, University of California, Santa Barbara, Santa Barbara, CA, United States

**Keywords:** California Conservation Genomics Project, CCGP, plant, reference genome, reciprocal translocation, ultramafic

## Abstract

The flowering plant genus *Aquilegia* (columbine) is an important contributor to biodiversity and an example of both biotic and abiotic niche adaptation across much of the Northern Hemisphere, especially in California. Here we report a near-chromosome level draft genome assembly for *A. eximia*, a California endemic species. *A. eximia* is a serpentine-soil specialist and is very closely related to 2 columbine species also being studied for the California Conservation Genomics Project (CCGP), *A. formosa* (widespread) and *A. pubescens* (high alpine). Utilizing high throughput, long reads (PacBio) and chromatin capture (Omni-C), the *A. eximia* genome makes marked contiguity improvements compared to the existing reference genome for another North American columbine, *A. coerulea* “Goldsmith.” The *A. eximia* genome will also be more useful for aligning whole genome resequencing data from California columbines than the genomes for more distantly related columbine species, the Asian *A. oxysepala* var. *kansuensis* and the European *A. vulgaris*. Notably, we found evidence that *A. eximia, A. coerulea* “Goldsmith,” and *A. vulgaris* all share the same overall genome structure and differ from *A. oxysepala* var. *kansuensis* by the same reciprocal translocation. The *A. eximia* reference genome will be a valuable tool for identifying patterns of plant biodiversity across California for the CCGP, as well as for future population genomic and trait mapping studies.

## Introduction

*Aquilegia* (columbines) is a genus of flowering plants of ca. 70 species (Munz 1946), having adapted to a wide variety of habitats and pollinators across the Northern Hemisphere over a short evolutionary timespan estimated at ca. 6 to 7 My (Whittall and Hodges 2007; Bastida et al. 2010; Fior et al. 2013; Zhai et al. 2019). In California, there are 3 predominant species, the widespread *A. formosa* Fish. ex DC. (Hook 1881), and 2 endemic species, *A. eximia* Van Houtte ex Planch (Planch 1857) and *A. pubescens*  (Coville 1893). Our goal here is to develop a high-quality reference genome that is suitable for assessing genetic diversity of all 3 species, allowing us to assess both a widespread species and those in extreme habitats.

**Fig. 1.**
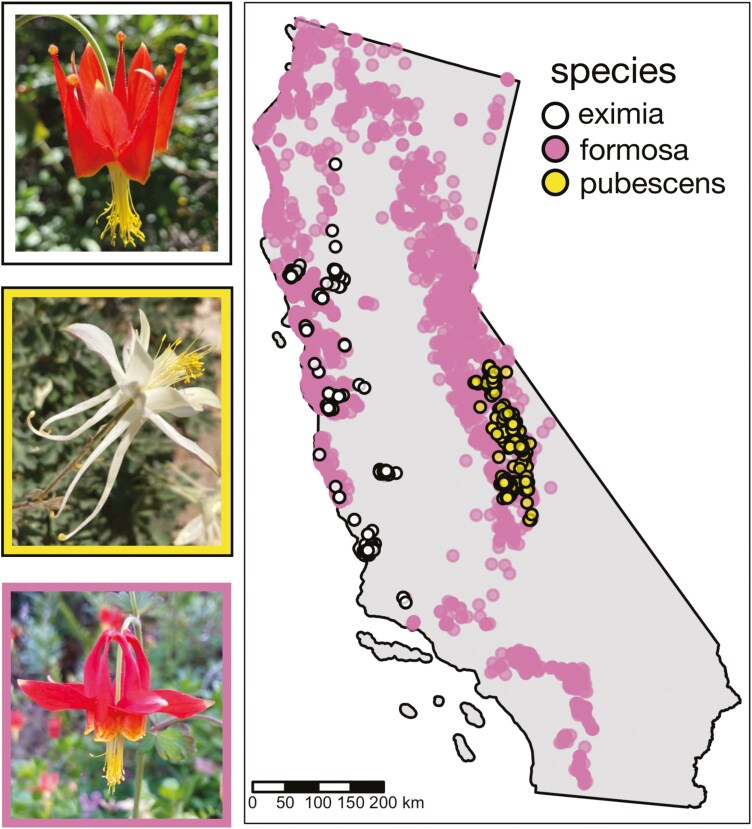
Photographs and observations of *Aquilegia* (columbine) species included in the CCGP study. Observation data are based on iNaturalist and personal collections. Border colors around photos correspond to the color of the dots on the range map.

*Aquilegia formosa* is found from northern Mexico to Alaska and occurs in 10/13 USDA ecoregions across the Southern California Mountains, Northern California Coast and Interior Coast Ranges, Klamath Range, the Southern, Central, and Northern California Coast, Sierra Nevada, Modoc Plateau, and the Northwest Basin and Range. This species has a very wide elevational distribution in California, from sea level to the tree line in the southern Sierra Nevada (~3,000 m), and encompasses the ranges of *A. eximia* and *A. pubescens* (Fig. 1). Thus, *A. formosa* is a good model for studying widespread California plant species, and the dynamics underlying their genetic variation across the landscape.

*Aquilegia eximia*’s range spans 6 USDA (United States Department of Agriculture) ecoregions along the central coastal mountains. The species is found almost exclusively in seeps crossing serpentine soil. Serpentine soils harbor just 1% of California’s land, but 12.5% of its endemic species, which have adapted to the shallow, nutrient poor, with high Mg/Ca and heavy metal-rich soils (Harrison et al. 2006). This species is likely derived from within *A. formosa*  (Whittall and Hodges 2007) and will be an important model for understanding the genetic connectivity across “islands” of serpentine habitat.

*Aquilegia pubescens* is also endemic to California and is found primarily above the tree line in 1 just USDA ecoregion, the southern Sierra Nevada mountains (Fig. 1). It is very closely related to *A. formosa* and *A. eximia* (Whittall and Hodges 2007) and forms natural hybrid zones with *A. formosa* where their distributions overlap (Hodges and Arnold 1994; Chase and Raven 1975; Grant 1976). This species is representative of high alpine species, allowing us to study connectivity and drivers of genetic diversity in this habitat threatened by climate change.

While there are 3 current genome assemblies for *Aquilegia*, each have compromises for an assessment of genetic diversity within and across species in California. The first genome assembly for the genus was from a horticultural derivative of a North American species, *A. coerulea* “Goldsmith,” henceforth referred to as *A. coerulea*. This genome was assembled prior to high-throughput long-read sequencing and proximity ligation technologies, using Sanger sequencing of a variety of plasmid insert sizes, mapping of F2 recombinant populations, and short-read Illumina sequences (Filiault et al. 2018). As such, the ordering of scaffolds within low recombination regions and high repeats such as pericentric regions was difficult. In fact, multiple QTL analyses of F2 populations using this genome to identify recombination breakpoints have required manual reordering of genomic blocks in these regions (Filiault et al. 2018; Ballerini et al. 2020; Edwards et al. 2021; Johns et al. 2024). A second genome assembly has been constructed for *A. oxysepala* var. *kansuensis*, which is a species from central Asia and substantially more distantly related from the California species. Alignment of resequencing data between species from different continents declines significantly (Filiault et al. 2018) making this genome a less useful reference for population genomic analyses in North American taxa. For example, comparison between the *A. coerulea* and *A. oxysepala* var. *kansuensis* genomes revealed a reciprocal translocation between chromosomes (Xie et al. 2020). The third previously assembled *Aquilegia* genome is for *A. vulgaris*, a species native to Europe and also relatively distantly related to North American species (NCBI accession GCA_964291885.1; Filiault et al. 2018). Thus, we chose to assemble a new genome sequence using the California endemic species, *A. eximia*. We chose to use *A. eximia* as its populations are usually small and isolated from 1 another, making inbreeding more likely, which would reduce heterozygosity and aid in genome assembly. All of these factors will make the *A. eximia* genome an important tool for aligning resequencing data for population genomic analyses such as the California Conservation Genomics Project (CCGP), as well as providing a more accurate reference genome for future population genomic and trait mapping studies (Shaffer et al. 2022).

## Methods

### Biological materials

We collected young leaves and floral tissue from a single individual grown from seed collected at Prefumo Canyon in San Luis Obispo County (34.417153, −119.842861). To collect tissue with reduced starch and secondary metabolites, the plant was placed in the dark for 3 to 4 days and then young expanding tissue was collected, weighed, snap frozen, and stored at −80 °C. We then returned the plant to the greenhouse to recover and produce more young tissue and repeated the collection cycle until we had a total of approximately 12 g of tissue.

### PacBio HiFi library preparation

High molecular weight (HMW) genomic DNA (gDNA) was extracted from 75 mg of leaves (PF Ex 6) using the Nanobind Plant Nuclei Big DNA Kit (Pacific BioSciences—PacBio, Menlo Park, CA) and (Workman et al. 2018) protocol with the following modification. We used nuclear isolation buffer supplemented with 350 mM Sorbitol to resuspend ground tissue and during the first wash of the nuclei pellet. The DNA purity was estimated using absorbance ratios (260/280 = 1.84 and 260/230 = 2.07) on the NanoDrop ND-1000 spectrophotometer. The final DNA yield (20 μg) was quantified using the Quantus Fluorometer (QuantiFluor ONE dsDNA Dye assay; Promega, Madison, WI). The size distribution of the HMW DNA was estimated using the Femto Pulse system (Agilent, Santa Clara, CA), and found that 70% of the fragments were 120 kb or more.

The HiFi SMRTbell library was constructed using the SMRTbell Express Template Prep Kit v2.0 (PacBio, Cat. No. 100-938-900) according to the manufacturer’s instructions. HMW gDNA was sheared to a target DNA size distribution between 15 and 18 kb. The sheared gDNA was concentrated using 0.45× of AMPure PB beads (PacBio, Cat. No. 100-265-900) for the removal of single-strand overhangs at 37 °C for 15 min, followed by further enzymatic steps of DNA damage repair at 37 °C for 30 min, end repair and A-tailing at 20 °C for 10 min and 65 °C for 30 min, and ligation of overhang adapter v3 at 20 °C for 60 min. The SMRTbell library was purified and concentrated with 1× AMPure PB beads (PacBio, Cat. No. 100-265-900) for nuclease treatment at 37 °C for 30 min and size selection using the BluePippin/PippinHT system (Sage Science, Beverly, MA; Cat. No. BLF7510/HPE7510) to collect fragments greater than 9 kb. The 15 – 20 kb average HiFi SMRTbell library was sequenced at UC Davis DNA Technologies Core (Davis, CA) using 0.5 8M SMRT cell, Sequel II sequencing chemistry 2.0, and 30-h movies on a PacBio Sequel IIe sequencer.

### Omni-C library preparation and sequencing

The Omni-C library was prepared using the Dovetail Omni-C Kit (Dovetail Genomics, Scotts Valley, CA) according to the manufacturer’s protocol with slight modifications. First, specimen tissue (leaves, ID: PF Ex 6) was thoroughly ground with a mortar and pestle while cooled with liquid nitrogen. Nuclear isolation was then performed using published methods (Workman et al. 2018). Subsequently, chromatin was fixed in place in the nucleus and digested under various conditions of DNase I until a suitable fragment length distribution of DNA molecules was obtained. Chromatin ends were repaired and ligated to a biotinylated bridge adapter followed by proximity ligation of adapter-containing ends. After proximity ligation, crosslinks were reversed, and the DNA was purified from proteins. Purified DNA was treated to remove biotin that was not internal to ligated fragments. An NGS library was generated using an NEB Ultra II DNA Library Prep kit (New England Biolabs, Ipswich, MA) with an illumine-compatible y-adaptor. Biotin-containing fragments were then captured using streptavidin beads. The post-capture product was split into 2 replicates prior to PCR enrichment to preserve library complexity with each replicate receiving unique dual indices. The library was sequenced at Vincent J. Coates Genomics Sequencing Lab (Berkeley, CA) on an Illumina NovaSeq 6000 platform to generate approximately 100 million 2 × 150 bp read pairs per gigabase of genome size.

### DNA sequencing and genome assembly

#### Nuclear genome assembly.

We assembled the genome of an *A. eximia* sample following the CCGP assembly pipeline V5.0, which uses PacBio HiFi reads and Omni-C data to produce high quality and highly contiguous genome assemblies. The pipeline is outlined in Table 1 and lists the tools and non-default parameters used in the assembly process. First, we removed the remnants adapter sequences from the PacBio HiFi dataset using HiFiAdapterFilt (Sim et al. 2022) and generated the initial phased diploid assembly using HiFiasm (Cheng et al. 2021, 2022) on Hi-C mode, with the filtered PacBio HiFi reads and the Omni-C dataset. We aligned the Omni-C data to both assemblies following the Arima Genomics Mapping Pipeline (https://github.com/ArimaGenomics/mapping_pipeline) and then scaffolded both assemblies with SALSA (Ghurye et al. 2017, 2019).

**Table 1.** Assembly pipeline and software used.

Assembly step	Software and any non-default options	Version	References
Initial assembly			
Filtering PacBio HiFi adapters	HiFiAdapterFilt	Commit 64d1c7b	Sim et al. (2022)
K-mer counting	Meryl (*k* = 21)	1	https://github.com/marbl/meryl
Estimation of genome size and heterozygosity	GenomeScope	2	Ranallo-Benavidez et al. (2020)
De novo *assembly (contiging)*	HiFiasm (Hi-C Mode, –primary, output hic.hap1.p_ctg, hic.hap2.p_ctg)	0.16.1-r375	Cheng et al. (2021) and Cheng et al. (2022)
Scaffolding			
Omni-C data alignment	Arima Genomics Mapping Pipeline	Commit 2e74ea4	https://github.com/ArimaGenomics/mapping_pipeline
Arima Genomics Mapping Pipeline (AGMP)	BWA-MEM	0.7.17-r1188	Li (2013)
	Samtools	1.11	Danecek et al. (2021)
	filter_five_end.pl (AGMP)	Commit 2e74ea4	https://github.com/ArimaGenomics/mapping_pipeline
	two_read_bam_combiner.pl (AGMP)	Commit 2e74ea4	https://github.com/ArimaGenomics/mapping_pipeline
	Picard	2.27.5	https://broadinstitute.github.io/picard/
Omni-C Scaffolding	SALSA (-DNASE, -i 20, -p yes)	2	Ghurye et al. (2017) and Ghurye et al. (2019)
Omni-C Contact map generation			
Short-read alignment	BWA-MEM (-5SP)	0.7.17-r1188	Li (2013)
SAM/BAM processing	Samtools	1.11	Danecek et al. (2021)
SAM/BAM filtering	Pairtools	0.3.0	Open2C et al. (2024)
Pairs indexing	Pairix	0.3.7	Lee et al (2022)
Matrix generation	Cooler	0.8.10	Abdennur and Mirny (2020)
Matrix balancing	hicExplorer (hicCorrectmatrix correct --filterThreshold -2 4)	3.6	Ramírez et al. (2018)
Contact map visualization	HiGlass	2.1.11	Kerpedjiev et al. (2018)
	PretextMap	0.1.4	https://github.com/wtsi-hpag/PretextView
	PretextView	0.1.5	https://github.com/wtsi-hpag/PretextMap
	PretextSnapshot	0.0.3	https://github.com/wtsi-hpag/PretextSnapshot
Manual curation tools	Rapid curation pipeline (Wellcome Trust Sanger Institute, Genome Reference Informatics Team)	Commit 7acf220c	https://gitlab.com/wtsi-grit/rapid-curation
Genome quality assessment			
Basic assembly metrics	QUAST (--est-ref-size)	5.0.2	Gurevich et al. (2013)
Assembly completeness	BUSCO (-m geno, -l embryophyta)	5.0.0	Manni et al. (2021)
	Merqury	2020-01-29	Rhie et al. (2020)
Contamination screening			
Local alignment tool	BLAST + (-db nt, -outfmt “6 qseqid staxids bitscore std,” -max_target_seqs 1, -max_hsps 1, -evalue 1e-25)	2.15	Camacho et al. (2009)
General contamination screening	BlobToolKit (HiFi coverage, BUSCO = embryophyta, NCBI Taxa ID = 1291435)	2.3.3	Challis et al. (2020)
Genome comparison			
Synteny plot	D-Genies (using minimap2, “hide noise”)	D-genies version 1.5.0 (minimap2 version 2) used in dgenies)	https://dgenies.toulouse.inra.fr/ Cabanettes and Klopp (2018) Li (2018)

Assemblies were manually curated by iteratively generating and analyzing their corresponding Omni-C contact maps. To generate the contact maps we aligned the Omni-C data with BWA-MEM (Li 2013), identified ligation junctions, and generated Omni-C pairs (Lee et al. 2022) using pairtools (Open2C et al. 2024). We generated a multi-resolution Omni-C matrix with cooler (Abdennur and Mirny 2020) and balanced it with hicExplorer (Ramírez et al. 2018). We used HiGlass (Kerpedjiev et al. 2018) and the PretextSuite (https://github.com/wtsi-hpag/PretextView; https://github.com/wtsi-hpag/PretextMap; https://github.com/wtsi-hpag/PretextSnapshot) to visualize the contact maps where we identified misassemblies and misjoins, and finally modified the assemblies using the Rapid Curation pipeline from the Wellcome Trust Sanger Institute, Genome Reference Informatics Team (https://gitlab.com/wtsi-grit/rapid-curation). Some of the remaining gaps (joins generated during scaffolding and/or curation) were closed using the PacBio HiFi reads and YAGCloser (https://github.com/merlyescalona/yagcloser). Finally, we checked for contamination using the BlobToolKit Framework (Challis et al. 2020).

#### Genome quality assessment.

We generated k-mer counts from the PacBio HiFi reads using meryl (https://github.com/marbl/meryl). The k-mer counts were then used in GenomeScope2.0 (Ranallo-Benavidez et al. 2020) to estimate genome features including genome size, heterozygosity, and repeat content. For contiguity metrics, we ran QUAST (Gurevich et al. 2013). To evaluate genome quality and functional completeness we used BUSCO (Manni et al. 2021) with the Embryophyta ortholog database (embryophyta_odb10) which contains 1,614 genes. Assessment of base level accuracy (QV) and k-mer completeness was performed using the previously generated meryl database and merqury (Rhie et al. 2020). We further estimated genome assembly accuracy via BUSCO gene set frameshift analysis using the pipeline described in (Korlach et al. 2017). Measurements of the size of the phased blocks is based on the size of the contigs generated by HiFiasm on HiC mode. We follow the quality metric nomenclature established by Rhie et al. (2021), with the genome quality code *x*·*y*·*P*·*Q*·*C*, where, *x* = log_10_[contig NG50]; *y* = log_10_[scaffold NG50]; *P* = log_10_ [phased block NG50]; *Q* = Phred base accuracy QV (quality value); *C* = % genome represented by the first “n” scaffolds, following a karyotype of 2n = 14, known for the number of chromosomes for this species (Genome on a Tree—GoaT; tax_tree [*A. eximia*; Challis et al. 2023]). Quality metrics for the notation were calculated on the assembly for the primary haplotype.

#### Comparison to other Aquilegia spp.

To assess broad synteny and genome structure we generated dot plots with D-Genies (Cabanettes and Klopp 2018) among 3 other publicly available genomes: *A. kansuensis* (Xie et al. 2020), *A. coerulea* (Filiault et al. 2018), and *A. vulgaris* (NCBI accession GCA_964291885.1). We used the 8 largest scaffolds of *A. eximia* to reduce complexity.

## Results

### Sequencing data

**Fig. 2.**
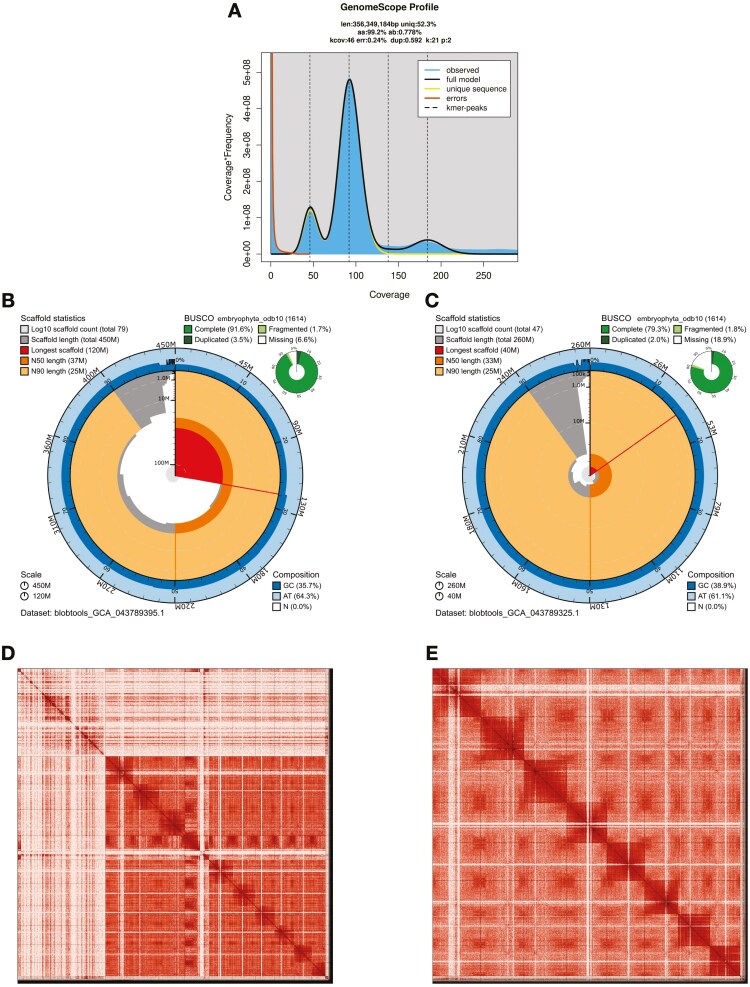
Overview of genome assembly metrics for Aquilegia eximia. a) K-mer spectrum output from PacBio HiFi data without adapters, generated using GenomeScope2.0. K-mers at lower coverage and lower frequency correspond to differences between haplotypes, whereas the higher coverage and higher frequency k-mers correspond to the similarities between haplotypes. B and c) Snail plots for the primary (b) and alternate (c) genome assembly showing a graphical representation of the quality metrics in Table 2 for the primary assembly. The circle represents the full size of the assembly. From the inside-out, the central plot displays length-related metrics. The red line represents the longest scaffold; other scaffolds are ordered by size moving clockwise around the plot and drawn in gray starting from the outside of the central plot. Dark and light orange arcs mark the scaffold N50 and N90 values. The central light gray spiral shows the cumulative scaffold count with a white line at each order of magnitude. White regions in this area reflect the proportion of Ns in the assembly The dark versus light blue area around it shows mean, maximum, and minimum GC versus AT content at 0.1% intervals (Challis et al. 2020). d and e) Hi-C Contact maps for the primary (d) and alternate (e) genome assembly generated with PretextSnapshot. Hi-C contact maps translate the proximity of genomic regions in 3-D space to contiguous linear organization. Each cell in the contact map corresponds to sequencing data supporting the linkage (or join) between 2 of such regions. Scaffolds numbers are indicated and are separated by black lines. Higher density corresponds to higher levels of fragmentation.

The Omni-C library generated 39.08 million read pairs and the PacBio SMRTBell library generated 802.07 thousand HiFi reads. The PacBio HiFi reads yielded ~29× genome coverage and had an N50 read length of 14,254 bp; a minimum read length of 123 bp; a mean read length of 13,508 bp; and a maximum read length of 46,837 bp (see Supplementary Fig. S1 for read length distribution). Based on the PacBio HiFi data, Genomescope 2.0 estimated a genome size of 367 Mb, a 0.559% heterozygosity rate and a 0.131% sequencing error rate. The k-mer spectrum shows a bimodal distribution with a major peak at ~29-fold coverage and a minor peak at ~15-fold coverage (Fig. 2a).

### Nuclear genome assembly

The final genome assembly (dmAquExim1) consists of 2 phased haplotypes, however, the assemblies have been randomly tagged as primary and alternate. Both assemblies are similar in size, with a difference of ~2 Mb between haplotypes. The final assemblies are also similar in size but not equal to the estimated genome assembly size from GenomeScope2.0, as it has been observed in other taxa (see Pflug et al. 2020, e.g.).

The primary haplotype (dmAquExim1.1.p) consists of 265 scaffolds spanning 363.4 Mb with a contig N50 of 6.09 Mb, a scaffold N50 of 47.38 Mb, the largest contig size of 20.83 Mb, and the largest scaffold size of 56.05 Mb. The alternate haplotype (dmAquExim1.1.a) consists of 185 scaffolds spanning 365.35 Mb with a contig N50 of 6.55 Mb, a scaffold N50 of 48.06 Mb, the largest contig size of 16.74 Mb, and the largest scaffold size of 55.6 Mb.

The primary haplotype has a BUSCO completeness score for the Embryophyta gene set of 97.8%, a base pair quality value (QV) of 61.85, a kmer completeness of 91.43%, and a frameshift indel QV of 50.83. The alternate haplotype has a BUSCO completeness score for the same gene set of 97.9%, a base pair QV of 62.95, a kmer completeness of 91.51%, and a frameshift indel QV of 50.32.

During manual curation we made a total of 167 joins (96 on the primary haplotype and 71 on the alternate haplotype) and 53 breaks (29 on the primary haplotype and 24 on the alternate haplotype) based on the signal from the Omni-C contact maps. We were able to close a total of 39 gaps, 20 on the primary haplotype and 19 on the alternate. No other contigs were modified or removed.

The Omni-C contact maps show highly contiguous assembly, suggesting that the *A. eximia* genome is organized in 8 major scaffolds based on the number of major bins along the diagonal axis of the plot, corresponding to 95.6% of the genome size (Fig. 2c and d). Assembly statistics are reported in Table 2 and represented graphically in Fig. 2c. We have deposited the genome assembly on NCBI GenBank (see Table 2 s details).

**Table 2.** Metrics for the primary and alternate assemblies of the *Aquilegia eximia* genome.

Bio Projects and vouchers	CCGP NCBI BioProject			PRJNA720569			
	Genera NCBI BioProject			PRJNA766267			
	Species NCBI BioProject			PRJNA808326			
	NCBI BioSample			SAMN25872407			
	Specimen identification			PFEx6			
	NCBI Genome accessions			Primary		Alternate	
	Assembly accession			JALIGU000000000		JALIGV000000000	
	Genome sequences			GCA_023053565.2		GCA_023053535.2	
Genome Sequence	PacBio HiFi reads		Run	1 PACBIO_SMRT (Sequel II) run: 802,141 spots, 10.8G bases, 6.6Gb			
			Accession	SRX15223256			
	Omni-C Illumina reads		Run	2 ILLUMINA (Illumina NovaSeq 6000) runs: 39.1M spots, 11.8G bases, 4Gb			
			Accession	SRX15223257, SRX15223258			
Genome Assembly Quality Metrics	Assembly identifier (Quality code)^a^				dmAquExim1(6.7.P6.Q61.C95)		
	HiFi Read coverage^b^			29.52X			
				Primary		Alternate	
	Number of contigs			382		281	
	Contig N50 (bp)			6,093,045		6,553,555	
	Contig NG50^b^			6,093,045		6,553,555	
	Longest Contigs			20,832,410		16,749,726	
	Number of scaffolds			265		185	
	Scaffold N50			47,382,029		48,063,828	
	Scaffold NG50^b^			47,382,029		48,063,828	
	Largest scaffold			56,061,779		55,609,086	
	Size of final assembly			363,407,297		365,358,954	
	Phased block NG50^b^			6,093,045		6,553,555	
	Gaps per Gbp (# Gaps)			322(117)		263(96)	
	Indel QV (Frame shift)			50.37125354		50.50182051	
	Base pair QV			61.85		62.95	
				Full assembly = 62.37			
	k-mer completeness			91.43		91.51	
				Full assembly = 98.46			
	BUSCO completeness (embryophyta) *n* = 1614		C^d^	S^d^	D^d^	F^d^	M^d^
		P^c^	98.02%	94.30%	3.72%	1.18%	0.81%
		A^c^	98.02%	94.30%	3.72%	1.12%	0.87%

^a^Assembly quality code x·y·P·Q·C derived notation, from Rhie et al. (2021). x = log10[contig NG50]; y = log10[scaffold NG50]; P = log10 [phased block NG50]; Q = Phred base accuracy QV (quality value); C = % genome represented by chromosomes as described in (Rhie et al. 2021), following a known karyotype for this species of 2n = 14 (Genome on a tree (query = Aquilegia eximia); Challis et al 2023). Quality code for all the assembly denoted by primary assembly (dmAquExim1.1.p).

^b^Read coverage and NGx statistics have been calculated based on the estimated genome size of 367 Mb.

^c^(P)rimary and (A)lternate assembly values.

^d^BUSCO Scores. Complete BUSCOs (C). Complete and single-copy BUSCOs (S). Complete and duplicated BUSCOs (D). Fragmented BUSCOs (F). Missing BUSCOs (M).

### Comparison with other *Aquilegia* genome assemblies

BUSCO analysis revealed high completeness of 97.8%, similar to the scores for the assemblies of *A. coerulea* (97.2%), *A. kansuensis* (97.9%), and *A. vulgaris* (98.3%). The estimated genome size, 367 Mb, is significantly larger than those found for the other 3 *Aquilegia* genome assemblies (Table 3).

**Table 3.** Assembly metrics for *Aquilegia* genomes. Numbers for *A. coerulea* and *A. kansuensis* come from Xie et al. (2020).

	Total size	# contigs	Contig N50	# scaffolds	Scaffold N50	BUSCO completeness	Sequencing technology
*A. eximia*	363 Mb	382	5.0 Mb	265	47.4 Mb	98.0%	PacBio, Omni-C
*A. coerulea*	292 Mb	7930	110 kb	1034	43.6 Mb	97.2%	Sanger, short-read Illumina
*A. kansuensis*	293 Mb	852	2.22 Mb	679	40.9 Mb	97.9%	Illumina, PacBio, BioNano, Hi-C
*A. vulgaris*	296 Mb	567	1.2Mb	134	42.7Mb	98.3%	PacBio, Arima2

BUSCO score represents complete BUSCOs.

**Fig. 3.**
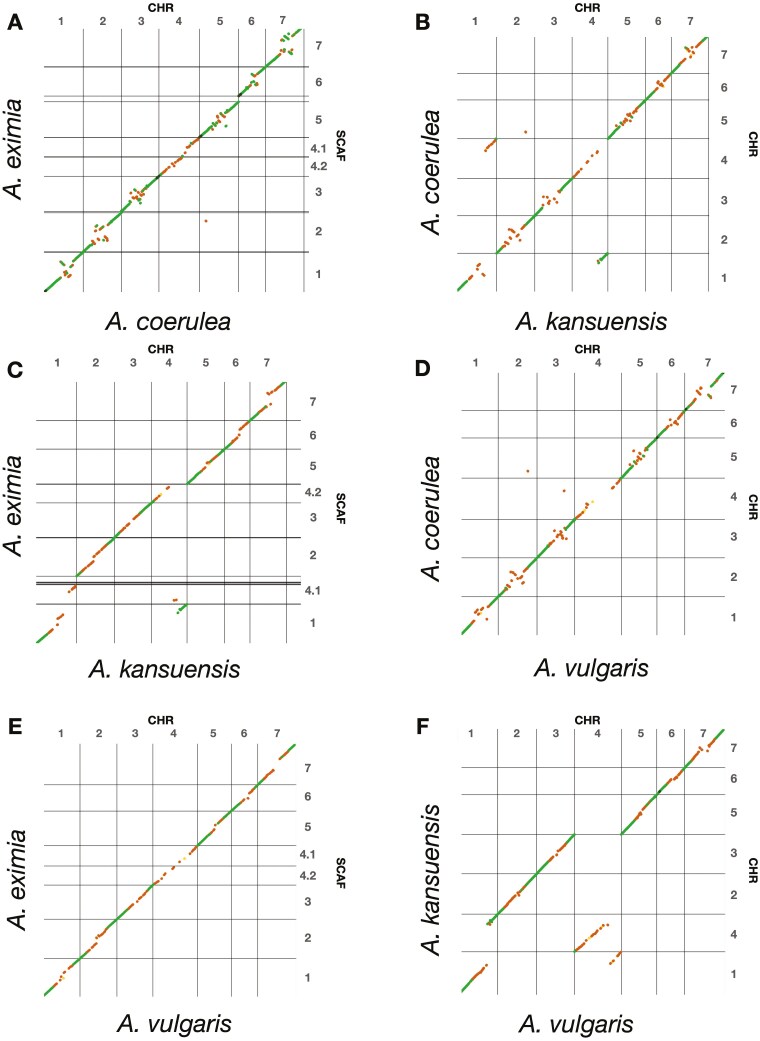
Dot plot alignments comparing *Aquilegia* genome assemblies: 7 chromosomes (labeled) for *A. coerulea, A. kansuensis*, and *A. vulgaris*, and the 8 largest scaffolds in *A. eximia* which are homologous with the 7 chromosomes in the other 3 species (labeled). We modified the chromosome numbers in *A. vulgaris* to match their homologs in *A. coerulea* and *A. kansuensis*.

Dot plots between *A. coerulea* and *A. kansuensis* reflected the results of (Xie et al. 2020), where the 7 chromosomes aligned to each other in large contiguous blocks of synteny except in presumably pericentromeric regions and an apparent reciprocal translocation between chromosomes 1 and 4 (Fig. 3a). In addition, in the pericentromeric regions, there are many smaller blocks of synteny but with scrambled relative orders and orientations. Comparing *A. eximia* to *A. coerulea*, *A. kansuensis*, and *A. vulgaris* revealed that the *A. eximia* assembly is near-chromosome level, where the largest 8 scaffolds of *A. eximia* were syntenic with the 7 chromosomes of the other species (Fig 3b and c). The alignment between *A. eximia* and *A. vulgaris* was relatively contiguous with no major rearrangements. Because the genome assembled into 8 large scaffolds rather than 7 as expected from the canonical chromosome number for the genus, we especially scrutinized the contact maps surrounding scaffolds 4.1 and 4.2, which align with chromosome 4 of *A. coerulea* and *A. vulgaris* (Fig. 2d and e and Supplementary Fig. S2). We found no convincing evidence from these data to merge scaffolds 4.1 and 4.2 into a single scaffold.

Notably, *A. eximia, A. coerula*, and *A. vulgaris* all share overall similar chromosome structures without any large translocations (Fig. 3a, d, and e). Specifically, all of scaffold 1 of *A. eximia* aligns with chromosome 1 of *A. coerulea* and *A. vulgaris*, and scaffolds 4.1 (JALIGU020000007.1) and 4.2 (JALIGU020000008.1) of *A. eximia* align to chromosome 4 of *A. coerulea* and *A. vulgaris*. However, when *A. eximia* is compared with *A. kansuensis*, the same reciprocal translocation between chromosomes 1 and 4 of *A. coerulea/A. kansusensis* was found (Fig. 3c). Furthermore, the *A. eximia* chromosomes show much higher contiguity in paracentric regions with *A. kansuensis* and *A. vulgaris* than with the more closely related *A. coerulea*. These 3 species, when compared to *A. coerulea*, each show scrambling of the relative orders and orientations of smaller synteny blocks in pericentromeric regions (Fig. 3a, b, and d) but when compared to each other, they show high contiguity (Fig. 3c, e, and f). This suggests that the ordering and orientation of scaffolds in the pericentromeric regions of the *A. coerulea* genome may be somewhat misplaced.

## Discussion

Here we present the first de novo reference genome for the California endemic, serpentine-adapted columbine, *A. eximia*. Three other genome assemblies are available for *Aquilegia* species and the *A. eximia* genome assembly has the highest contig N50 at 5.0 Mb (compared to *A. kansuensis* at 2.2 Mb, *A. vulgaris* at 1.2 Mb, and *A. coerulea* at 110 kb) as well as the highest scaffold N50 at 47.4 Mb (compared to *A. coerulea* at 43.6 Mb, *A. vulgaris* at 42.7 Mb, and *A. kansuensis* at 40.9 Mb; Filiault et al. 2018; Xie et al. 2020). The *A. eximia* genome assembly also has a BUSCO completeness score that is very high (98.0%), similar to other *Aquilegia* genome assemblies (Table 3). Lastly, the *A. eximia* genome is significantly larger, spanning 363 Mb, compared to genome sizes of 292 to 296 Mb for *A. coerulea*, *A. kansuensis*, and *A. vulgaris*. This larger genome size is reflected across each of the 7 chromosomes (counting *A. eximia* scaffolds 4.1 and 4.2 together when comparing to chromosome 4 of the other species).

We further compared the structure of *Aquilegia* genome assemblies using dot plots and found, overall, very similar structures. The most striking difference is that the *A. eximia* assembles into 8 large scaffolds (26.3 to 56.0 Mb) while the 3 other *Aquilegia* genomes each assemble into 7 large scaffolds. The canonical karyotype of *Aquilegia* species is *n* = 7 (Gregory 1941; Taylor 1967) although there are at least 7 species with reports of karyotypes of both *n* = 7 and *n* = 8 (Dawe and Murray 1981; Subramanian 1985; Chung et al. 2013). The *A. eximia* scaffolds 4.1 and 4.2 align with chromosome 4 of *A. coerulea* and *A. vulgaris* (Fig. 3a and e) suggesting that either chromosome 4 has split into 2 chromosomes in *A. eximia* or that our data are insufficient to merge these 2 scaffolds together (Supplementary Fig. S2). Future studies of karyotypes, recombination maps and FISH analysis would be useful in resolving these possibilities. In addition, it is interesting that chromosome 4 is involved with this difference in scaffold numbers because chromosome 4 has many enigmatic characteristics compared to the other chromosomes such as a higher rate of polymorphism, lower gene density, lower gene expression, as well as harboring the 5S and 35S rDNA repeats (Filiault et al. 2018). As previously reported, *A. kansuensis* differs in chromosome structure from *A. coerulea* due to a reciprocal translocation between chromosomes 1 and 4 (Xie et al. 2020) and our data shows that *A. eximia* shares the structure of *A. coerulea* as does *A. vulgaris* (Fig. 3a and e). Lastly, overall, the *A. eximia* genome shows much more contiguous alignments, especially in paracentric regions, with *A. kansuensis* and *A. vulgaris* than with *A. coerulea* (Fig. 3). This is likely due to mis-assembly of the *A. coerulea* genome, which is the only assembly that lacked Hi-C data.

Given the high contiguity and completeness of the *A. eximia* genome, it will be an excellent reference genome for population genetic studies and genetic mapping studies, especially for closely related North American taxa. Furthermore, it has an especially close phylogenetic relationship with *A. formosa* and *A. pubescens*. Thus, it is a strong asset for the goals of the CCGP and for studying plant adaptation to some of California’s most important contributors to biodiversity, such as serpentine soil, high alpine, and riparian corridors.

## Supplementary material

Supplementary material is available at *Journal of Heredity* online.

esaf035_suppl_Supplementary_Figures_S1-S2

## Data Availability

Data generated for this study are available under NCBI BioProject PRJNA808326. Raw sequencing data for sample AexPC6 (NCBI BioSample SAMN25872407) are deposited in the NCBI Short Read Archive (SRA) under SRR19156777 for PacBio HiFi sequencing data, and SRR19156775-6 for the Omni-C Illumina sequencing data. GenBank accessions for both primary and alternate assemblies are GCA_023053565.2 and GCA_023053535.2; and for genome sequences JALIGU000000000 and JALIGV000000000. Assembly scripts and other data for the analyses presented can be found at the following GitHub repository: www.github.com/ccgproject/ccgp_assembly.
