# Supplementary material for: A near chromosome-level assembly of the serpentine endemic columbine, Aquilegia eximia
Source: J Hered. 2025 Jun 10;117(1):85–95. doi: 10.1093/jhered/esaf035 (PMC12767194; doi:10.1093/jhered/esaf035)
Supplement: esaf035_suppl_Supplementary_Figures_S1-S2 [file esaf035_suppl_supplementary_figures_s1-s2.pdf]

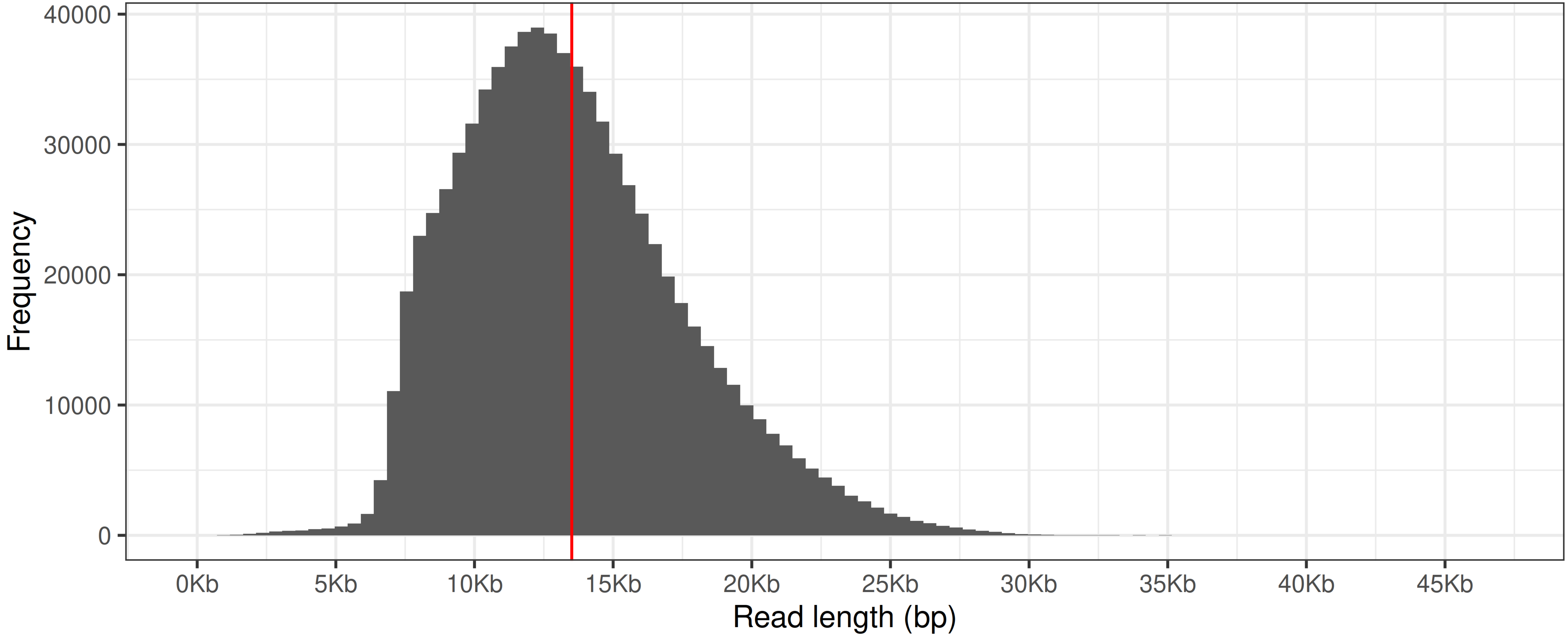

Supplementary Figure S1. Histogram of PacBio HiFi read lengths

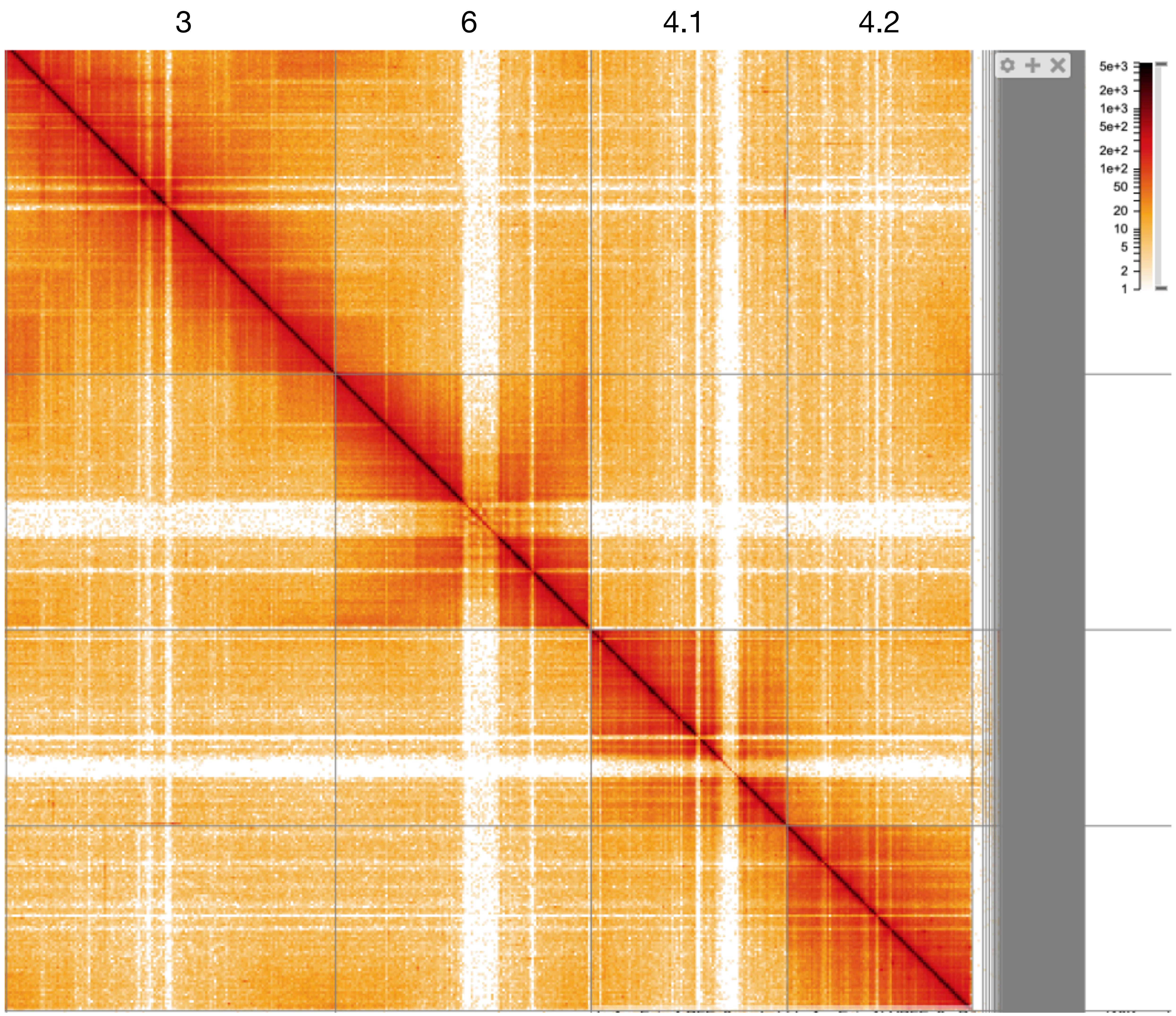

Supplementary Figure S2. Zoomed in Hi-C contact map of scaffolds 4.1 and 4.2, as well as the two neighboring scaffolds.
